# Robust Object Recognition under Partial Occlusions Using NMF

**DOI:** 10.1155/2008/857453

**Published:** 2008-05-15

**Authors:** Daniel Soukup, Ivan Bajla

**Affiliations:** Smart systems division, ARC Seibersdorf research GmbH, 2444 Seibersdorf, Austria

## Abstract

In recent years, nonnegative matrix factorization (NMF) methods of a reduced image data representation attracted the attention of computer vision community. These methods are considered as a convenient part-based representation of image data for recognition tasks with occluded objects. A novel modification in NMF recognition tasks is proposed which utilizes the matrix sparseness control introduced by Hoyer. We have analyzed the influence of sparseness on recognition rates (RRs) for various dimensions of subspaces generated for two image databases, ORL face database, and USPS handwritten digit database. We have studied the behavior of four types of distances between a projected unknown image object and feature vectors in NMF subspaces generated for training data. One of these metrics also is a novelty we proposed. In the recognition phase, partial occlusions in the test images have been modeled by putting two randomly large, randomly positioned black rectangles into each test image.

## 1. Introduction

Subspace methods represent a separate branch of high-dimensional data analysis, such as in areas of computer vision and pattern recognition. In particular, these methods have found efficient applications in the fields of face identification and recognition of digits and characters. In general, they are characterized by learning a set of basis vectors from a set of suitable image templates. The subspace spanned by this vector basis captures the essential structure of the input data. Having found the subspace (offline phase), the classification of a new image (online phase) is accomplished by projecting it on the subspace in some way and by finding the nearest neighbor of templates projected onto this subspace.

In 1999, Lee and Seung [1] showed for the first time that for a collection of face images an approximative representation by basis vectors, encoding the mouth, nose, and eyes, can be obtained using a nonnegative matrix factorization (NMF). NMF is a method for generating a linear representation of data using nonnegativity constraints on the basis vector components and the coefficients. It can formally be described as follows:

(1) V ≈W⋅H ,

where **V** ∈ *ℛ* ^*n* × *m*^ is a positive image data matrix with *n* pixels and *m* image sample templates (template images are usually represented in lexicographic order of pixels as column vectors), **W** ∈ *ℛ* ^*n* × *r*^ are reduced *r* basis column vectors of an NMF subspace, and **H** ∈ *ℛ* ^*r* × *m*^ contains coefficients of the linear combinations of the basis vectors needed to reconstruct the original data. Usually, *r* is chosen by the user so that (*n* + *m*)*r* < *n* *m*. Then each column of the matrix **W** represents a basis vector of the generated (NMF)subspace. Each column of **H** represents the weights needed to approximate the corresponding column in **V** (image template) by means of the vector basis **W**. Various error functions were proposed for NMF, such as in the papers of Lee and Seung [2] or Paatero and Tapper [3].

The main idea of NMF application in visual object recognition is that the NMF algorithm identifies localized parts describing the structure of that object type. These localized parts can be added in a purely additive way with varying combination coefficients to form the individual objects. The original algorithm of Lee and Seung could not achieve this locality of essential object parts in a proper way. Thus other authors investigated the possibilities to control the sparseness of the basis images (columns in **W**) and the coefficients (matrix **H**). The first attempts consisted in altering the norm that measures the approximation accuracy, like LNMF [4, 5]. Hoyer introduced a method for steering the sparsenesses of both factor matrices, **W** and **H**, with two sparseness parameters [6, 7]. In their work, Pascual-Montano et al. briefly summarized and described all NMF algorithms used in this topic [8]. Their approach also led to a sparseness control parameter, but only one for both matrices. The optimization algorithm remained equal to the one already introduced by Lee and Seung.

One important problem by using NMF for recognition tasks is how to obtain NMF subspace projections for new image data that are comparable with the feature vectors determined in NMF coded in matrix **H**. Guillamet and Vitrià [9] propose one method in their work that consists of rerunning the NMF algorithm for new image data keeping **W** constant. However, in the conventional method, training images and new images are orthogonally projected onto the determined subspace. Both methods have advantages and drawbacks. We will discuss them in more detail and propose a modification of the NMF task that comprises the advantages of both methods.

An important aspect in measuring distances in NMF subspaces, which is necessary in recognition tasks, is the used metric. NMF subspace basis vectors do not form an orthogonal system. Due to this fact, it is not convenient to apply the natural Euclidean metric. Guillamet and Vitrià [9] experimented with several alternative metrics: *L*1, *L*2, cos, and EMD. They lined out that solely EMD takes the positive aspects of NMF into account. As this metric is computationally demanding, Ling and Okada [10] proposed a new dissimilarity measure, the diffusion concept, which is as accurate as EMD, but computationally much more efficient. Liu et al. [11, 12] proposed to replace the Euclidean distance in NMF recognition tasks by a weighted Euclidean distance (a version of Riemannian distance). These authors also experimented with orthogonalized bases. However, as commented by authors, these modified NMF bases are not part-based anymore.

In our research, we focus on studying the influence of matrix sparseness parameters, subspace dimension, and the use of distance measures on the recognition rates, in particular for partially occluded objects. We use Hoyer's algorithms to achieve sparseness control. Additionally, we propose a modification of the entire NMF task similar to the methods of Yuan and Oja [13] and Ding et al. [14]. The implementation of our modification additionally comprises Hoyer's sparseness control mechanisms. In the case of studying proper distance measures, we propose a new metric.

In Section 2, we briefly review Hoyer's method (Section 2.1). Section 2.2 contains a presentation of the motivation and a detailed description of our modification of the NMF task. Section 2.3 is about distance measuring in NMF subspaces. We present the metrics we used for our experiments and propose the anew distance measure. Then we present the setup and results of our experiments in Section 3. Section 4 contains conclusions and a future outlook.

## 2. NMF with Sparseness Constraints

The aim of the work of Hoyer [7] is to constrain NMF to find a solution with prescribed degrees of sparseness of the matrices **W** and **H**. The author claims that the balance of the sparseness between these two matrices depends on the specific application and no general recommendation can be given. The modified NMF problem and its solution is given by Hoyer as follows.

### 2.1. Hoyer's Method---Nmfsc

#### 2.1.1. Problem Definition

Given a nonnegative data matrix **V** of size *n* × *m*, find the nonnegative matrices **W** and **H** of sizes *n* × *r* and *r* × *m* (resp.,) such that

(2) $$E(W,\;H)=\;∥V-WH{∥}^{2}$$

is minimized, under optional constraints

(3) $$\begin{array}{cc} s(w_{i}) & =s_{W},\;\forall_{i},\;\;i=1,\ldots ,r,\\ s(h_{i}) & =s_{H},\;\forall_{i},\;\;i=1,\ldots ,r, \end{array}$$

where **w**_*i*_ is the *i*th *column of* **W**, **h**_*i*_ *is the* *i*th row of **H**. Here, *r* denotes the dimensionality of an NMF subspace spanned by the column vectors of the matrix **W**, and *s*_*W*_ and *s*_*H*_ are their desired sparseness values. The sparseness criteria proposed by Hoyer [7] use a measure based on the relationship between *L*_1_ and *L*_2_ norm of the given vectors **w**_*i*_ or **h**_*i*_. In general, for the give *n*-dimensional vector **x** with the components *x*_*i*_, its sparseness measure *s* (**x**) is defined by the formula:

(4) $$s(x):=\frac{\sqrt{n}-L_{1}/L_{2}}{\sqrt{n}-1}=\frac{\sqrt{n}-\sum \left|x_{i}\right|/\sqrt{\sum x_{i}^{2}}}{\sqrt{n}-1}\;.$$

This measure quantifies how much energy of the vector is packed into a few components. This function evaluates to 1 if and only if the given vector contains a single nonzero component. Its value is 0 if and only if all components are equal. It should be noted that the scales of the vectors **w**_*i*_ or **h**_*i*_ have not been constrained yet. However, since **w**_*i*_⋅**h**_*i*_ = (**w**_*i*_ *λ*)⋅(**h**_*i*_/*λ*), we are free to arbitrarily fix any norm of either one. In Hoyer's algorithm, the *L*_2_ norm of **h**_*i*_ is fixed to unity.

#### 2.1.2. Factorization Algorithm

The projected gradient descent algorithm for NMF with sparseness constraints essentially takes a step in the direction of the negative gradient, and subsequently projects onto the constraint space, making sure that the taken step is small enough that the objective function is reduced at every step. The main muscle of the algorithm is the projection operator proposed by Hoyer [7], which enforces the required degree of sparseness.

### 2.2. Modified NMF Concept: modNMF

In the papers mentioned up to now, the attention was concentrated on methodological aspects of NMF as a part-based representation of image data, as well as on numerical properties of the developed optimization algorithms applied to the matrix factorization problem. It turned out that the notion of matrix sparseness involved in NMF plays the central role in part-based representation. However, little effort has been devoted to systematic analysis of the behavior of the NMF algorithms in actual pattern recognition problems, especially for partially occluded data.

**Figure 1:**
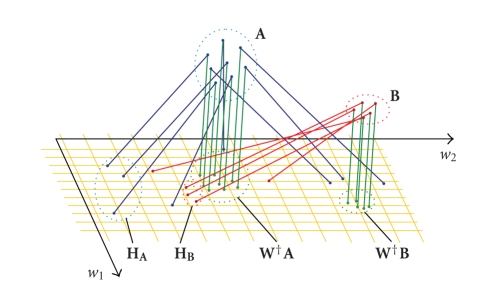
Visualization of the Nmfsc results for a low-dimension example (3D data sets A and B as training points). The plane spanned by *w*_1_ and *w*_2_ represents the NMF subspace due to this training set. **H**_**A**_ and **H**_**B**_ are the training set projections to the subspace implicitly given by matrix **H** in the NMF algorithm. **W** ^†^ **A** and **W** ^†^ **B** are the orthogonal projections of the training sets onto the NMF subspace.

For a particular recognition, task of objects represented by a set of training images (**V**) we need: (i) to calculate in advance (in an offline mode) projection vectors of the training images onto the obtained vector basis (**W**)—the so-called feature vectors—, and then (ii) to calculate (in an online mode) a projection vector onto the obtained vector basis (**W**) for each unknown input vector **y**. Guillamet and Vitrià [9] propose to use the feature vectors determined in the NMF run, that is, columns of matrix **H**. The problem of determining projected vectors for new input vectors in a way that they are comparable with the feature vectors is solved by the authors by rerunning the NMF algorithm. In this second run, they keep the basis matrix **W** constant and the matrix **V**_test_ contains the new input vectors instead of the training image vectors. The results of the second run are the searched projected vectors in the matrix **H**_test_. However, this method has some drawbacks. We investigated the function of NMF exemplarily for 3D point data instead of high-dimensional images. These points have been divided into two classes based on point proximity. The two classes are called **A** and **B** and are illustrated in Figure 1. We ran NMF to get a two dimensional subspace visualized as yellow grid in Figure 1 spanned by the two vectors *w*_1_ and *w*_2_, which together build matrix **W**. Additionally, we show the feature vectors of the input point sets (**H**_**A**_ and **H**_**B**_ in Figure 1) and connected each input point with its corresponding feature vector in the subspace plane (projection rays). Especially for the point set A, it can be observed that the projection rays are all nonorthogonal, with respect to, the plane and that their mutual angles significantly differ (even for feature vectors belonging to the same class). Thus the feature vectors of set A and set B are not separated clusters anymore. We have doubts that a reliable classification based on proximity of feature vectors is achievable in this case. A second possibility to determine proper feature vectors for an NMF subspace, which is conventionally used (e.g., mentioned by Buciu [15]), is to recompute the training feature vectors for the classification phase entirely new by orthogonally projecting the training points (images) onto to NMF subspace. Unknown input data to be classified are similarly orthogonally projected to the subspace. This method is also visualized in Figure 1: from each input point an orthogonal dotted line is drawn to the orthogonal projections of the points into the subspace plane. It can be noticed that the feature vectors determined in this way preserve a separation of the feature vector clusters, corresponding to the cluster separation in the original data space (point sets **W** ^†^ **A** and **W** ^†^ **B**). In view of these observations, we propose to favor the orthogonal projection method.

Nonetheless, both methods have their disadvantages. The method of Guillamet and Vitria operates with nonorthogonal projected feature vectors that directly stem from the NMF algorithm and do not reflect the data cluster separation in the subspace. On the other hand, the conventional method does not accommodate the optimal data approximation result determined in NMF because one of the two optimal factor matrices is substituted by a different one in the classification phase. Our intention was to combine the benefits of both methods into one, that is, benefits of orthogonal projections of input data and preservation of the optimal training data approximation of NMF. We achieve this by changing the NMF task itself. Before we present this modification, we recall in more detail how the orthogonal projections of the input data are computed.

As the basis matrix **W** is rectangular, matrix inversion is not defined. Therefore, one has to use a pseudo-inverse of **W** to multiply it from the left onto **V** (cf. [15]). Orthogonal projections of data points *y* onto a subspace defined by a basis vector matrix **W** are realized by solving the following overdetermined equation system:

(5) W b=y

for the coefficient vector **b**. This can, for instance, be achieved via the Moore-Penrose (M-P) pseudoinverse (This may not be the numerically stable way, but in our investigations we could not observe differences to other usually more appropriate methods.) **W** ^†^ giving the result for the projection as

(6) $$b=W^{†}\;y.$$

Similarly, for the NMF feature vectors (in the offline mode) we determine **H**_LS_ = **W** ^†^ **V**, where **H**_LS_ are projection coefficients obtained in the least squares (LS) manner. These coefficients can differ severely from the NMF feature vectors implicitly given by **H** (see Figure 1). It is important to state that the entries of **H**_LS_ are not nonnegative anymore, **H**_LS_ also contains negative values.

If one has decided to use the orthogonal projections of input data onto the subspace as feature vectors, the fact that the matrix **H** is not used anymore in the classification phase and that the used substitute for **H**—**H**_LS_—is not nonnegative anymore, gives rise to the questions whether matrix **H** is necessary at all in NMF and whether corresponding coefficient coding necessarily has to be nonnegative. Moreover, using the orthogonal projection method, we do not make use of the optimal factorization achieved by NMF, as the coefficient matrix is altered for classification. Consequently, we propose the following modification of the NMF task itself:

*given the training matrix* **V**, *we search for a matrix* **W** *such that*

(7) $$V\approx W(W^{†}V).$$

Within this novel concept (*modNMF*), **W** is updated in the same way as in common NMF algorithms. Even the sparseness of **W** can be controlled by the standard mechanisms, for example, those of Hoyer's method. Only the coding matrix **H** is substituted by the matrix **W** ^†^ **V** to determine the current approximation error. Thus this new concept can be applied to all existing NMF algorithms. In our research, we implemented and analyzed modNMF comprising the sparseness control mechanisms of Hoyer.

There are two existing methods that are related to modNMF in two complementary ways. In projective NMF (*pNMF*) of Yuan and Oja [13], the independent factor matrix **H** is given up and, similarly to modNMF, substituted by a coding matrix derived from **W** and **V**, namely, **W** ^*T*^ **V**. Ding et al. [14] realize the second change incorporated in modNMF. They keep two independent factor matrices in their *semi-NMF* method, but give up the nonnegativity restriction for one of them. Unlike modNMF, the nonnegativity constraint is kept for the coding matrix **H** while the signs in the subspace basis **W** are not restricted. Following Ding et al. notion, modNMF is also only semi-nonnegative. The resulting subspaces of semi-NMF and modNMF are not classical NMF subspaces anymore. However, in the traditional NMF methods, as we have outlined above, the training images have to be orthogonally projected to the determined subspace in preparation of the object recognition phase and this also results in mixed sign subspace vectors. (Due to this very fact that for traditional NMF methods as well as for modNMF the actually used subspace features in the recognition phase are not purely nonnegative and to the fact that the determined subspace bases are all nonorthogonal in general, we face the same problems in the recognition phase for modNMF and classical NMF subspaces. To simplify matters in this paper, we summarize all these subspaces in the notion *NMF subspaces* in our further discussions, which address the issues related to recognition experiments in such subspaces.) The difference in the case of modNMF is that the orthogonal projections of the training images onto the subspace that are used in the recognition task (**W** ^†^ **V**) are also those for which the factorization that optimally approximates the training data **V** is achieved. In semi-NMF, this is not guaranteed, that is, extra orthogonal projection of the training images onto the subspace has to be done to prepare an object recognition phase. These extra projections do not comprise the structure of the optimally approximating factor matrix determined in the factorization run, just like in classical NMF methods. Similarly to modNMF, pNMF assures that the subspace features are the orthogonal projections of the training images onto the subspace, while these very subspace features simultaneously constitute the optimal factorization matrix in the sense of approximating **V**. Actually, pNMF is in some sense a special case of modNMF. Both try to optimize **W** with the goal to approximate an identity matrix as close as possible in form of the factor in front of *V*-modNMF in the case of **W** **W** ^†^ and pNMF for **W** **W** ^*T*^. Thus although orthogonality of **W** in pNMF may not be explicitly demanded, within the factorization process, **W** has to approximate an orthogonal matrix more and more as the approximation improves. Thanks to the fact that the more general modNMF model does not contain such structural restrictions on **W** (except nonnegativity), there are more degrees of freedom in modNMF to approximate **V** accurately. Moreover, the sparseness of **W** can be controlled in modNMF via the sparseness parameter.

### 2.3. Distances in NMF Subspaces

Having solved the NMF task for the given training images (matrix **V**), the vector basis of an NMF subspace (of the original data space) is generated as columns of the matrix **W**. Depending on the sparseness of **W** and **H** controlled in the algorithms, the basis vectors in **W** manifest different mutual angles, that is, the basis is not orthogonal. With increasing sparseness of **W** or decreasing sparseness of **H**, the mutual angles tend to be closer to orthogonality. If both sparseness parameters are adjusted, dependence on them is not so obvious and straightforward.

As outlined by various authors mentioned in Section 1, suitable metrics for measuring the distances of NMF subspace points have to be defined, due to the non-orthogonality of NMF subspace bases. In our work, we compared the four metrics *Euclidean*, *diffusion*, *Riemannian*, and *ARC-distance*.

For comparison reasons, we also included the Euclidean metric (*d* ^2^(*x*_1_, *x*_2_) = (*x*_1_ − *x*_2_)^*T*^(*x*_1_ − *x*_2_)), which is commonly supposed not to be suitable in vector spaces with nonorthogonal basis. The diffusion distance is derived from the EMD metric, for which Guillamet and Vitrià [9] argued that it is well suited to the positive aspects of NMF. The complete derivative of the diffusion distance can be found in the work of Ling and Okada, who developed this dissimilarity concept to achieve a computationally more efficient algorithm.

The third metric, Riemannian distance, will be described in more detail, as it is the basis of our proposal, ARC-distance. Liu and Zheng [11] defined the Riemannian distance as a weighted Euclidean distance as *d*_*G*_^2^(*x*_1_, *x*_2_) = (*x*_1_ − *x*_2_)^*T*^ **G**(*x*_1_ − *x*_2_), where **G** is a similarity matrix defined as **G** = **W** ^*T*^⋅**W**. They claimed that adopting this Riemannian metric is more suitable than the Euclidean distance for classification when using nearest neighbor classifiers.

For the standard Euclidean metric *d* ^2^ and Riemannian metric *d*_*G*_^2^ of two vectors *x*, *y* from a subspace, the following formulas can be drawn: *d*_*G*_^2^(*x*, *y*) = (*x* − *y*)^*T*^ **W** ^*T*^ **W**(*x* − *y*) = (**W**(*x* − *y*))^*T*^ **W**(*x* − *y*) = *d* ^2^(**W** *x*, **W** *y*). This proves that the Riemannian distance measures the Euclidean distance of the back-projected subspace vectors, that is, the subspace points represented in the orthogonal image super space bases. Thus the Riemannian distance takes the angle structure of the NMF subspace bases into account.

To be able to deal with partial occlusions, the correctly chosen distance measure should also be able to discriminate two specific cases of vectors: (i) a case for which the value of the Riemannian distance of two vectors is large because of great deviations in all components of these vectors, and (ii) a case when only a few components contribute to the great value of the Riemannian distance, that is, when the error of recognition is sparsely distributed over the feature vector components. Therefore, to define a modified Riemannian (shortly “ARC-distance”) distance, we introduce a sparseness term into the Riemannian metric formula, that is, *d*_*G*_^2^(*x*, *y*) = (*x* − *y*)^*T*^ **G** (*x* − *y*)(1 − **s**(|*x* − *y*|)), where **s** measures the sparseness (compare Section 2) of the absolute difference of the feature vectors. Note that the sparseness should be measured in the feature space, as each component in this space representation is optimized to reflect one essential part of the training image objects.

## 3. Results of Computer Experiments

The goal of our study was to investigate influences of *sparseness control parameters* and *subspace metrics* on recognition rates of unoccluded and occluded images. In massive computer experiments, we have varied the dimensions of the NMF subspaces from *r* = 25 up to *r* = 250, similarly to the papers of Guillamet and Vitrià [9] and Liu and Zheng [11]. The method of *nearest neighbor classification* has been used for object recognition.

**Figure 2:**
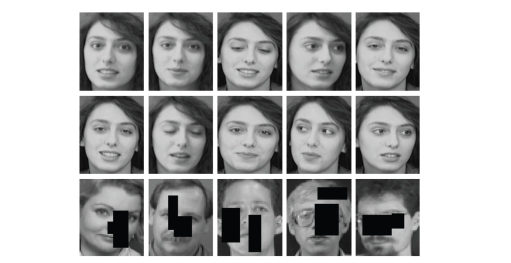
An example of face images of one person selected from the ORL face database—two top lines. An example of different randomly occluded faces—the bottom line.

**Figure 3:**
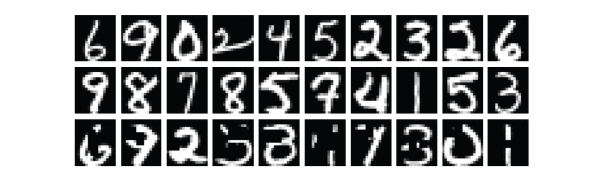
An example of handwritten digit images selected from the USPS database—two top lines. An example of different randomly occluded digits—the bottom line.

**Figure 4:**
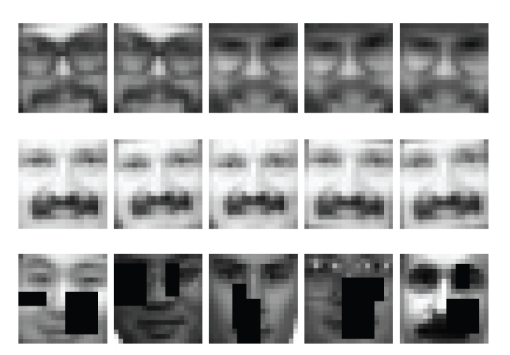
An example of face images of two persons selected from the CBCL face database—two top lines. An example of different randomly occluded faces—the bottom line.

For our experiments, we chose three widely used image databases: (i) the Cambridge ORL face database (cited in paper of Li et al. [5]; grey-level images with resolution 92 × 112, which were down sampled for our experiments to the size 46 × 58 = 2668 pixels) and (ii) USPS handwritten digit database (cited in the paper of Liu and Zheng [11]; grey-level images with resolution 16 × 16 = 256 pixels), and (iii) CBCL image database available at the web address: http://cbcl.mit.edu/cbcl/software-datasets/FaceData2.html (cited in the paper of Hoyer [7]) that contains grey-level face images with resolution 19 × 19 = 361 pixels. We simulated object occlusions in test images as two rectangles of random (but limited) sizes with random super positioning on an original image (see Figures 2, 3, and 4).

In the case of ORL database, the number of training images was 222, and the number of testing images was 151. These two sets of images were chosen as disjunctive sets. For the experiments with USPS database, we chose 2000 training images and 1000 testing images (different from the training ones again). (In the USPS recognition rate plots (Figures 6, 8), data points for *r* = 175 are missing. This is due to a Matlab problem that could not be solved. For some reason, all subspace files containing matrix **H** with dimension 175 × 2007 were corrupted and could not be opened anymore. We were able to reproduce the error in simplified configurations, however, we were not able to solve it. As the recognition curves do not oscillate a lot, we found it justified to just interpolate between the two neighboring points of the point in *r* = 175.) For the case of the CBCL image database, we used 1620 training images and 809 testing images.

### 3.1. Nmfsc---Unoccluded versus Occluded Test Images

**Figure 5:**
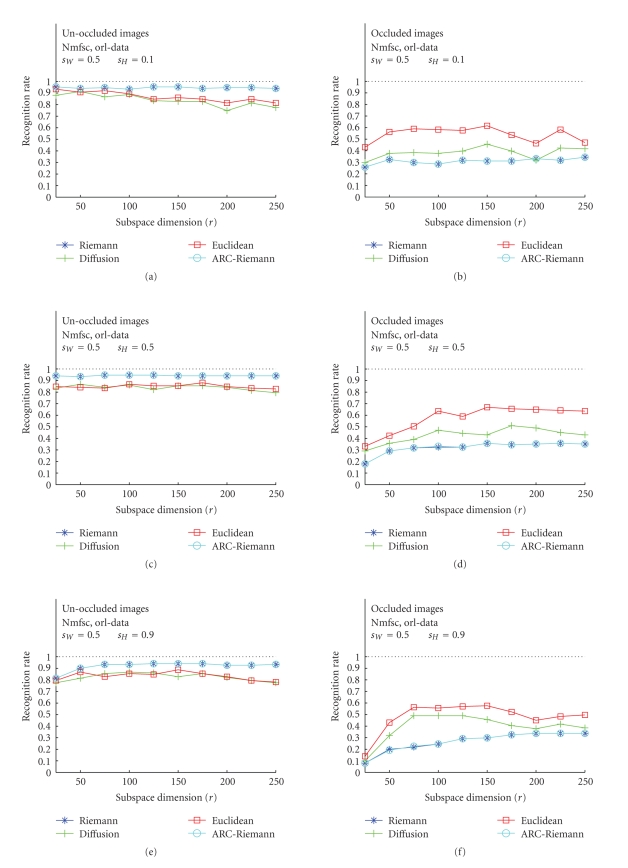
Classification results for ORL training image data using Hoyer's method. (a), (c), (e): unoccluded test images for *s*_*W*_ = 0.5, *s*_*H*_ = 0.1, 0.5, 0.9. (b), (d), (f): occluded test images for the identical values of the sparseness parameters.

**Figure 6:**
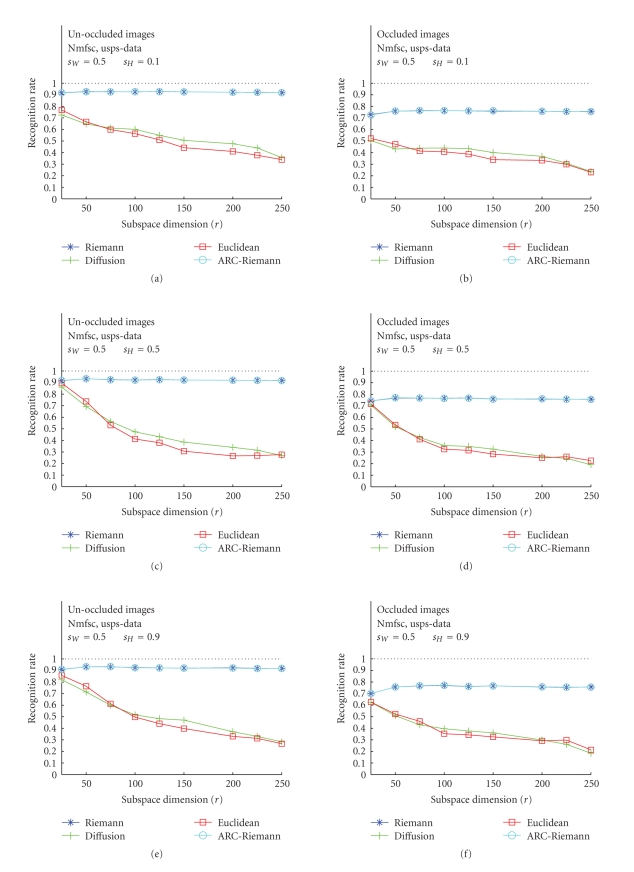
Classification results for USPS training image data using Hoyer's method. (a), (c), (e): unoccluded test images for *s*_*W*_ = 0.5, *s*_*H*_ = 0.1, 0.5, 0.9. (b), (d), (f): occluded test images for the identical values of the sparseness parameters.

**Figure 7:**
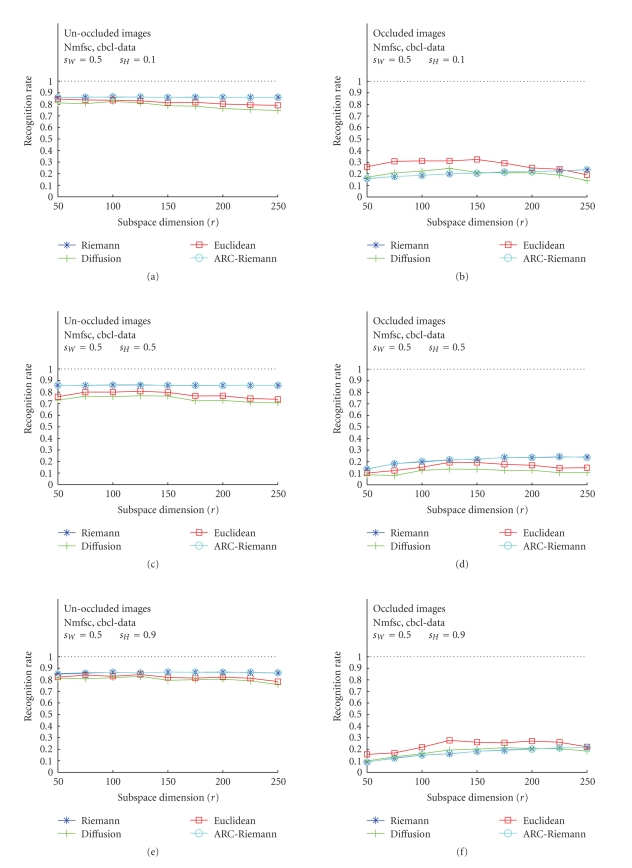
Classification results for CBCL training image data using Hoyer's method. (a), (c), (e): unoccluded test images for *s*_*W*_ = 0.5, *s*_*H*_ = 0.1, 0.5, 0.9. (b), (d), (f): occluded test images for the identical values of the sparseness parameters.

The results of the first set of our experiments, accomplished for all three image bases, and for unoccluded, as well as occluded images are displayed in Figures 5, 6, and 7. The acronym “Nmfsc” stands here for Hoyer's NMF method with coded sparseness (*s*_*W*_, *s*_*H*_). In this set of tests, Hoyer's Nmfsc-algorithm was applied consecutively to ORL face images, USPS digits, and CBCL face images. The algorithms have been trained for various combinations of sparseness parameter values. The resulting NMF subspaces, calculated for different dimensions *r* = 25, 50,…, 250 were used for recognition experiments. We used four types of distances to measure the distance of each projected test image to the nearest feature vector (of the templates) in the given subspace. For each NMF subspace, a recognition rate (RR) over all test images was calculated. The plots show RR versus subspace dimension *r* (unoccluded—(a), (c), (e), and occluded—(b), (d), (f)). The plots with the best recognition results have been chosen.

For unoccluded images, all three data sets show similar RR behavior in the cases of the Riemannian-like metrics (Riemannian and ARC-distance), only CBCL RR are slightly smaller. The Euclidean and diffusion curves for the ORL and CBCL data are almost as high as for the Riemannian-like measures, but also, as one would expect. Their behavior for USPS data even more fulfills these expectations, as they are much smaller than the Riemannian-like RR curves and, moreover, decrease with increasing dimension. This behavior is expectable, as more (nonorthogonal) basis vectors introduce more error components into the distance computation. This happens due to the fact that Euclidean and diffusion distance do not take into account the mutual basis vector angles. The dimension reduction for all datasets is very high, as for Riemannian-like metric all three achieve the maximal RR at about *r* = 50. Remarkable is that ARC-distance does not differ from Riemannian distance. It can be seen (Figures 7(a), 7(c), 7(e)) that the RR values for all types of distances are lower (below 0.9) than those achieved for ORL faces. There are only small differences in RR between the cases corresponding to application of different distances, but in general, Riemannian distance yields the maximum values.

The RR behavior for occluded data differs severely between ORL and USPS data. First, RR maxima for USPS data are higher than for ORL data—below 0.7 in the ORL case versus about 0.75 for USPS data. Second, for ORL data the RR curves of the metrics do not behave in the expected way. Euclidean and diffusion distance generate much better results than the Riemannian-like. For USPS, RR behave qualitatively in the same way as in the unoccluded case, RR values are only smaller. Finally, RR maxima are achieved for higher dimension values in the ORL case, that is, a much smaller dimension reduction. In the case of CBCL image database, the situation changes dramatically in comparison to that of ORL face images: in average the RR are 50% smaller, they are reaching approximately the value of 0.3 (comparing to 0.7 maximum for ORL). For two value combinations of the sparseness parameters in Hoyer's method (Figures 7(b), 7(f)) the Euclidean distance yields higher RR, though it is not strictly monotone; however, for the case D, the Riemannian distance outperforms Euclidean and diffusion ones. The difference between RR values for Riemannian distances on one side, and Euclidean and diffusion distances on the other side are apparent but not so large as is the case of ORL face images.

### 3.2. Occluded Test Images---Nmfsc versus modNMF

**Figure 8:**
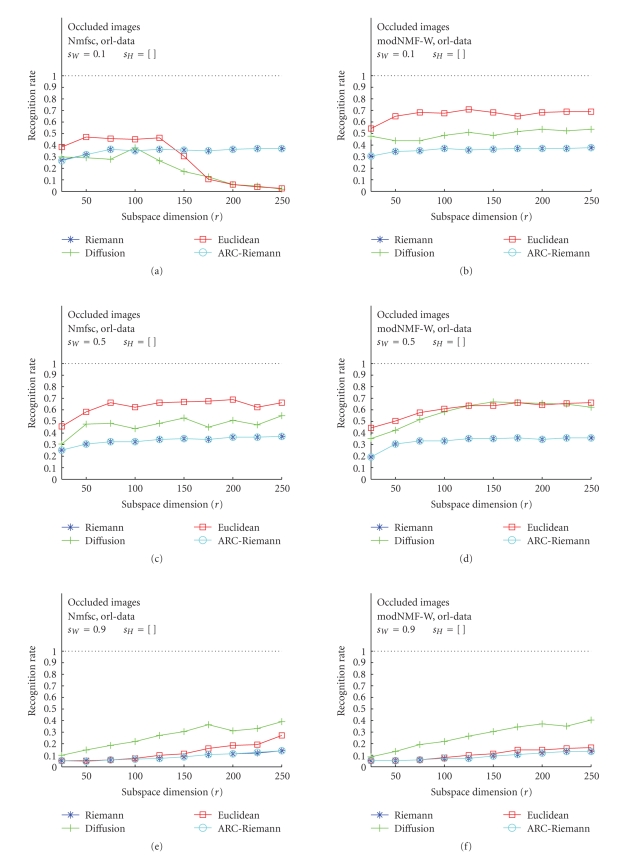
Classification results for ORL training image data. (a), (c), (e): Hoyer's Nmfsc algorithm applied to occluded test images for *s*_*W*_ = 0.1,0.5,0.9, *s*_*H*_ = [ ]. (b), (d), (f): our modified modNMF algorithm applied to occluded test images for the identical values of the sparseness parameters.

**Figure 9:**
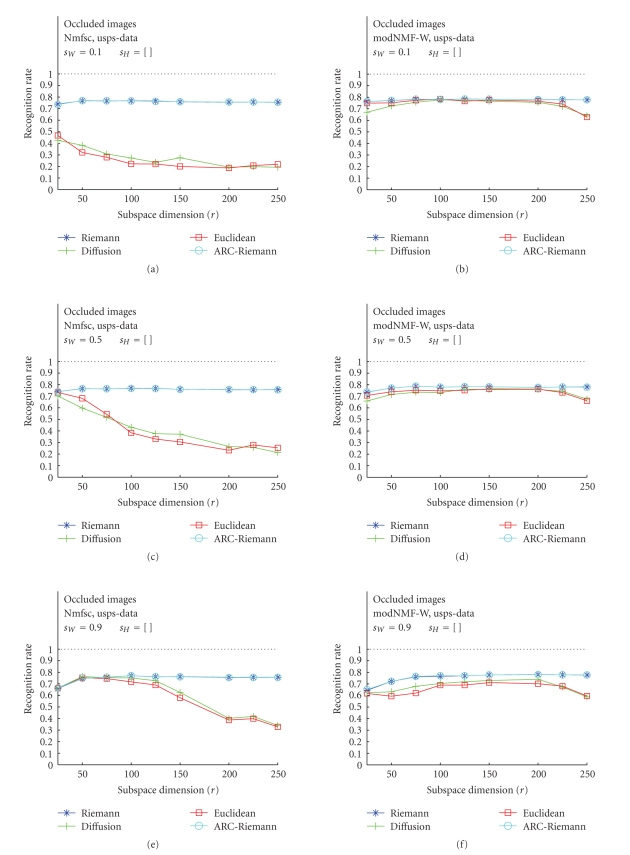
Classification results for USPS training image data. (a), (c), (e): Hoyer's Nmfsc algorithm applied to occluded test images for *s*_*W*_ = 0.1, 0.5, 0.9, *s*_*H*_ = [ ]. (b), (d), (f): our modified modNMF algorithm applied to occluded test images for the identical values of the sparseness parameters.

**Figure 10:**
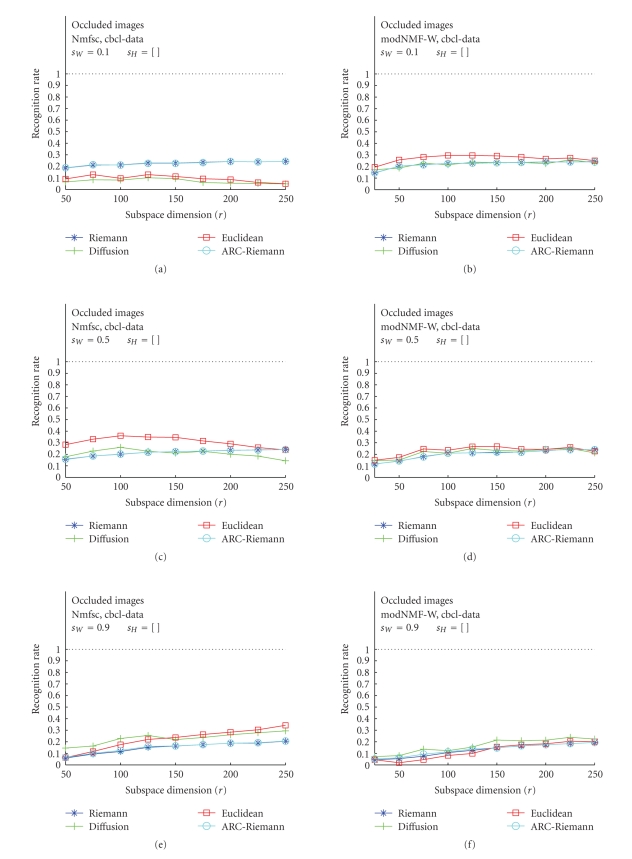
Classification results for CBCL training image data. (a), (c), (e): Hoyer's Nmfsc algorithm applied to occluded test images for *s*_*W*_ = 0.1, 0.5, 0.9, *s*_*H*_ = [ ]. (b), (d), (f): our modified modNMF algorithm applied to occluded test images for the identical values of the sparseness parameters.

In the second part of our study, we were interested in a comparison of the RR of Nmfsc and modNMF, latter one being implemented with Hoyer's sparseness control mechanisms. Of course, since the NMF methodology is intended mainly to generate part-based subspace representation of template images, our further interest was concentrated only on occluded images. These results, obtained for optimum values of sparseness parameter *s*_*W*_, are displayed in Figures 8, 9, and 10. The plots also show RR versus subspace dimension *r* but the columns now discriminate the used algorithms (Nmfsc—(a), (c), (e), and modNMF—(b), (d), (f)). The plots with the best recognition results have been chosen.

The qualitative behavior of the RR curves of ORL faces according to the distance measures is the same as described in Section 3.1. Euclidean and diffusion distances unexpectedly dominate the riemannian-like metrics. Except a break-in of RR values for the Euclidean and diffusion distances in the case of Nmfsc with *s*_*W*_ = 0.1, both algorithms, Nmfsc and modNMF achieve approximately the same results (Figure 8). The qualitatively more expected and quantitatively better results (w.r.t., RR maxima) are obtained in the case of the USPS data. For Nmfsc with only the *s*_*W*_ parameter set, the Riemannian-like RR curves dominate the Euclidean and diffusion distances, whereas—as expectable—the latter decrease with increasing dimension and decreasing sparseness *s*_*W*_ (Section 2.3). Remarkable is that the novel modNMF algorithm increases and stabilizes the performance of the Euclidean and diffusion distances. The plots show that the curves of these two metrics are close to the Riemannian-like ones. The CBCL image data comprise face images which have significantly lower spatial resolution than the face data in the ORL image base, while the structure of their parts is similarly complex. These characteristics are reflected in apparent decrease of recognition rates for occluded images for both methods being compared. In general, the behavior of the recognition rates manifests in this case very low sensitivity to the choice of the sparseness parameters. None of the distances applied exhibits unique prevalence.

## 4. Conclusions

In this paper, we have analyzed the influence of the matrix sparseness, controlled in NMF tasks via Hoyer's algorithm [7], from the viewpoint of object recognition efficiency. A special interest was devoted to partially occluded images, since images without occlusions can similarly well be handled by all NMF methods. Besides, Hoyer's algorithm, we introduced a modified version of the NMF concept—modNMF—using a term containing the Moore-Penrose pseudoinverse of the basis matrix **W** instead of the coefficient matrix **H**. Among the discussed important theoretical advantages, this method provides the computational benefit that the subspace projections of the training images do not have to be calculated after subspace generation in an additional step. The novel concept was implemented comprising the sparseness modification mechanism of Nmfsc. A further goal of the paper was to analyze and compare RR achieved for four different metrics used in the recognition tasks. As NMF subspace bases are nonorthogonal, distance measuring is a crucial aspect. The computer experiments were accomplished for three different image databases, ORL, USPS, and CBCL. In the classification tasks, we used the nearest neighbor method. In the unoccluded cases, Riemannian-like distances dominate RR quality in maxima and stability over all subspace dimensions and all parameter settings. ORL and USPS only differ slightly in the behavior of Euclidean and diffusion distances. In the case of CBCL, small differences of RR are manifested between the cases using different distances. The conclusions related to the results for the occluded test images can be summarized as follows.

(1) The ability of NMF methods to solve recognition tasks is dependent on the kind of used images and the databases as a whole. Independently of the method, the RR for USPS data are higher than those for ORL face data. This finding could be ascribed to the simpler structure of the digits (almost binary data, lower resolution, objects sparsely cover the image area). Moreover, USPS contain much larger classes (USPS: 2000 training images for only 10 classes, ORL: 222 images with only 5 training images per class), so that the interclass variations in USPS can better be covered. In general, the RR obtained for faces from the CBCL database are significantly worse than in comparable cases with ORL face images. We assign these results to the poor resolution of the structured face image data.

(2) Not following the overall expectation, Euclidean and diffusion distances showed better recognition performances for occluded test images in the case of ORL data. As these do not take into account subspace bases angles this is a surprise. USPS data treated with Hoyer's Nmfsc method behave like expected: with increasing dimension and decreasing*s*_*W*_ (i.e., increasing orthogonality, see Section 2.3), the RR measured with Euclidean and diffusion distances decrease (almost) monotonically. On the other hand, using our modNMF method, Euclidean and diffusion distances perform almost as well as the Riemannian-like metrics overall dimensions and sparseness values. This gives a hint that the relatively bad performances of these two metrics for the Nmfsc method cannot totally be ascribed to the nonorthogonality of the bases, but to the used orthogonal projections of the training images (**H**_LS_) instead of the well approximating factor matrix **H** (**V** ≈ **W**⋅**H**) in the classification phase; since we have observed no differences between the RR for the original Riemannian distance and ARC-distance, the proposed formula will need further exploration, likely to introduce some kind of numerical emphasis of the added sparseness term, as for example, exponential.

(3) Massive recognition experiments using Nmsfc and modNMF algorithms, reported in our preliminary study [16], showed minor influence of sparseness parameter *s*_*H*_ on recognition rates in cases of unoccluded, as well as occluded images selected from three mentioned image databases. Therefore, in the recognition experiments with occluded images included in this study, the sparseness parameter *s*_*H*_ has not been controlled and we have been experimenting exclusively with sparseness value *s*_*W*_ of the NMF basis matrix. Namely, we used three representative values: *s*_*W*_ = 0.1, 0.5, 0.9. As mentioned above we applied two NMF methods, conventional Nmsfc and our modified modNMF algorithm. Based on the analysis of the plots of RR for these methods and for images from three image databases, given in Figures 8, 9, and 10, the following conclusions on influence of the sparseness *s*_*W*_ on RR can be drawn as follows:

- *ORL face images*: *Nmsfc method*: the maximum RR have been achieved for *s*_*W*_ = 0.5, the minimum RR have been achieved for *s*_*W*_ = 0.9; *modNMF method*: the maximum RR have been obtained for *s*_*W*_ = 0.1, however, the values of RR for *s*_*W*_ = 0.5 were close to maxima; the minimum values of the RR have been obtained for *s*_*W*_ = 0.9;
- *CBCL face images*: *Nmsfc method*: the maximum RR have been achieved for *s*_*W*_ = 0.5, the minimum RR have been achieved for *s*_*W*_ = 0.1; *modNMF method*: the maximum RR have been obtained for *s*_*W*_ = 0.1, however the values of RR for *s*_*W*_ = 0.5 were, similarly to the case of ORL, also close to maxima; the minimum values of the RR have been obtained for *s*_*W*_ = 0.9;
- *USPS digit images*: for both NMF methods compared, there were no significant influence of the sparseness parameter *s*_*W*_ on RR observed.

USPS performed better and followed the overall expectations better than ORL and CBCL. We basically ascribe this fact to the different training data situations. As mentioned in the first point above, inter-class variations were much more covered for the USPS dataset than for the face images. The novel modNMF algorithm even improved the results achieved in the case of the already well performing USPS data set. ARC-distance in its current form did not fulfill the expectations in the experiments. Significantly, lower spatial resolution of the CBCL face data than the face data in the ORL image base is reflected in apparent decrease of recognition rates for occluded images for both methods being compared. Various distances used for the CBCL database manifested little influence on RR.

Spratling [17] analyzed the methodological situation related to the concept of “part-based” representation of image data by NMF subspaces, and pointed on the weaknesses of application of this concept in the NMF framework. Inspired by Spratling's results, we have analyzed possibilities of further research of improvement of the NMF methodology using a revisited version of this concept that could be more attractive for object recognition tasks with occlusions. The research into this NMF version is in progress.
